# Gender-dependent Differences in the Relationship between Diabetes Mellitus and Ambient Air Pollution among Adults in South Korean Cities

**Published:** 2017-03

**Authors:** Dongwook SOHN, Hyunjin OH

**Affiliations:** 1. Dept. of Architectural Engineering, Yonsei University, Seoul, South Korea; 2. Dept. of Nursing, Gachon University, Incheon, South Korea

**Keywords:** Air pollution, Diabetes, Gender specificity, PM_10_, SO_2_

## Abstract

**Background:**

Air pollution has been a serious public health threat worldwide. It has been linked to pulmonary and cardiovascular diseases but is also believed to contribute to air-pollution-mediated cardiometabolic disease such as diabetes. We investigated the relationship between type 2 diabetes mellitus (DM2) and air pollution in densely developed urban settings in South Korea, using national epidemiologic data.

**Methods:**

The analysis focused on examining gender-related differences in the relationship between DM 2 and air pollutants, specifically particulate matter ≤ 10μm (PM_10_) and sulfur dioxide (SO_2_). To assess the relationship between DM and exposure to PM_10_ and SO_2_, multivariate logistic regression models were developed using the 2012 Korea Community Health Survey data and the ambient air pollution data in South Korean cities at both *Gu*- and *Si* levels.

**Results:**

The commonly encountered levels of PM_10_ and SO_2_ may be associated with DM 2 prevalence in South Korea but it appears there may be gender differences. In particular, exposure to either PM_10_ or SO_2_ was significantly related to the prevalence of DM 2 among women but not among men.

**Conclusion:**

These findings provide new evidence of an association between air pollution and the risk of diabetes in urbanized areas of South Korea.

## Introduction

Air pollution is a serious public health threat worldwide, particularly in developed and newly urbanizing countries (1, 2). Exposure to relatively high levels of air pollution is associated with myocardial infarction (3), arrhythmias (4) and endothelial dysfunction (5). The suspected link between urbanization and concomitant dietary factors and inactivity has been studied for its effect on the development of chronic diseases. Beyond the contribution of both dietary and activity patterns, exposure to environmental pollutants found in urban areas may independently explain in part the link between urbanization and the emergence of cardiometabolic diseases such as type 2 diabetes mellitus (DM2). To date, the association between air pollution and diabetes remains speculative due largely to limitations in research design. While progress has occurred, a great deal of research is still needed. It should focus on this association and the complicated circumstances behind the relationship between air pollution and risks for DM.

DM shares many risk factors with cardiovascular diseases (6), so suspected to be associated with exposure to air pollution (7). Systematic reviews and meta-analyses based on observational and longitudinal cohort data confirm that exposure to air pollution may be associated with the incidence or prevalence of DM among urbanized populations (8–10). The results of meta-analyses substantiate the possibility that both gaseous pollutants and particulate matter (PM) increase the risk of DM (8). Specifically, long-term exposure to particulate matter ≤ 2.5μm (PM_2.5_), particulate matter ≤ 10μm (PM_10_), and nitrogen dioxide (NO_2_) is significantly related to the incidence of DM (9). DM is an outcome of exposure to air pollution, and metabolic detoxification genes affect air pollution outcomes (11). Their study examined the associations between air pollutants and markers of insulin resistance (IR) and the effect modification by GSTM1, GSTT1, and GSTP1 genotypes among elderly participants in the Korean Elderly Environmental Panel (KEEP) study. PM_10_ and NO_2_ might increase IR in the elderly.

Recent evidence on the relationships between DM and air pollution shows that this association may differ by gender. For example, traffic-related air pollution was associated with DM prevalence in women, but not in men (12, 13). The gender-dependent effect of PM exposure on insulin resistance also leads to an increased risk of DM in women (14). Gender differences may exist and could be associated with physiological differences in inflammatory responses or with lifestyle and activity patterns in men and women (6).

Contrary to this evidence and speculation about the link between air pollution, gender, and DM, the results of other studies do not support a link between gender, DM and air pollution (15, 16). Such contradictions and inconsistencies across study findings raise several issues and point to important design flaws. Limitations in research methodology have been stated to be present due to a wide variety of research challenges including. However, not limited to: poor research design (observational and retrospective versus prospective designs), sample bias or limits in sample size, lack of objective diagnostic data, pollution exposure measurement errors and data lacking to support the biological plausibility of a link between air pollution and diabetes (6, 8).

Together with these limitations is the fact that few studies have been conducted across urbanized countries. Air pollution and the prevalence of diabetes has been and is an ever growing concern in other populations such as Asia and the Middle East but there is limited available data from non-western populations (8, 17). Recently some progress has been made with new groundbreaking studies out of Asia and the Middle East. The recent longitudinal study of elderly Koreans in the Korean Elderly Environmental Panel (KEEP) evaluated the associations between air pollutants and markers of insulin resistance (IR). Air pollutants (namely of PM_10_, O_3_, and NO_2_) may increase IR among elderly population (11). Another study in the Middle East explored the link between type 2 DM and air pollution using the geographical distribution of air quality index in one air-polluted city. In this study, no significant associations were found between air pollution and the prevalence of diabetes (18).

The purpose of the present study was to investigate the relationship between the prevalence of DM and air pollution in densely developed urban settings in South Korea, using cross-sectional nationwide epidemiologic data. The study built on findings derived from nationwide data repositories versus small observational studies. Further, our analyses examined the suspected linkages between air pollution, gender, and DM. We specifically focused on examining gender-related differences in the relationship between DM and two air pollutants: PM_10_ and SO_2_ in South Korean cities.

## Methods

### Study Design

This study used the Korea Community Health Survey (KCHS), 2012 of adult residents living in urban areas of South Korea for which the ambient air pollution data at the finest grain (*Gu* and *Si* levels) were available. *Gu* refers to the administrative subdivision of a metropolitan city in South Korea which has a population of more than a half million people. A *Gu* has an average size of approximately 50km^2^; *Si* is one of the subdivisions of a province, which has a population of more than 150000 people. The study area included 84 *Gu*s and 39 *Si*s located in urban areas nationwide.

Institutional Review Board of Gachon University as a minimal risk study (1044396-201501-HR-003-01) approved the study.

### Samples

The primary data source was responses from residents of the Korea Community Health Survey (KCHS) 2012. The KCHS is a nationwide cross-sectional health interview survey conducted by the Korea Centers for Disease Control and Prevention (KCDC). The original survey consisted of questions on personal health-related behaviors including health problems, health status, and disease condition in Korean neighborhoods (19). From 229226 survey respondents, we excluded those living in rural areas (n=99467) and those with missing data regarding both the survey questions on health-related behaviors and the measurements of ambient air pollution (n=33691). A final 96068 samples were selected within the spatial boundary of the study area (the administrative areas of 84 *Gu*s and 39 *Si*s) from the full set of KCHS data. The prevalence of DM among the study population was 6.2% (n=5911). DM was assessed by a self-reported questionnaire during the interview. Since KCHS was conducted using individual-level participant interviews, DM as an outcome measurement in this study was based on the participants’ reports. Among the participants, those with DM or were taking anti-diabetic medications were classified as subjects with type 2 DM.

### Study Measures

Approval for using the survey data was given from the KCHS. The KCHS is a national surveillance system that has monitored the health and nutritional status of Korean residents since 2008 (19). This nationally representative cross-sectional survey uses trained interviewers to collect information on participants’ socioeconomic status, health-related behaviors, quality of life, healthcare utilization, anthropometric measures, biochemical and clinical profiles for non-communicable diseases, and dietary intake. The survey consists of five components: health behavior, vaccination, disease, health care service, accident/addiction, and quality of life (19).

### Measurement of Population Characteristics

Data about age, household income, and economic status were directly obtained from the KCHS. Household income is a continuous variable that estimates the participant’s annual household income. Economic activity status is a dichotomous variable that describes whether the participant is currently working. Body mass index (BMI=weight (kg) / height^2^ (m^2^)) is estimated based on the participants’ health screening. The measure of educational attainment was categorized into three groups: elementary school or less, middle to high school, and some college or higher level of education. The variable of smoking behavior was categorized into four groups: non-smoker, former smoker, occasional smoker, and daily smoker.

### Measurement of Ambient Air Pollution

The annual average of the concentration of two air pollutants, PM_10_ and SO_2_, in the survey participants’ neighborhoods was estimated using *Gu* and *Si*-level air pollution data that were obtained from the Korean Statistical Information Service (KOSIS). The positive effect of PM_10_ on the prevalence of DM has been reported in studies conducted in North America and Europe (8) examined in the analysis. With China’s extensive use of coal fuels as the primary energy source, SO_2_ is considered one of the cross-border air pollutants that have a major impact on the air quality in Korea (20). Hence, SO_2_ from China will continue to have a great influence on Korea’s air pollution (21), the potential effect of SO_2_ on DM prevalence was also analyzed.

Concerning epidemiologic studies investigating the health effects of air pollution, it is particularly important to reduce the measurement errors for the exposure to air pollution (22, 23). Specific research methods, such as limiting the degree of error through careful data collection, making adjustments for measurement error in statistical analyses, and minimizing the spatial unit of data collection, can control the measurement error (22, 24). The present study attempted to reduce the exposure measurement error by using air pollution data at the finest grain (*Gu* and *Si*-level) available in South Korea. The data provided information on monthly average concentration levels of air pollutants measured at the monitoring stations located in *Gu*s and *Si*s in the country. The annual averages of PM_10_ and SO_2_ concentration in the survey participants’ neighborhoods were estimated and used in the logistic regression.

### Data Analyses

To assess the relationship between the annual mean of the PM_10_ and SO_2_ exposure levels and DM2, multivariate logistic regression models were developed using the 2012 KCHS data and the ambient air pollution data of urban areas at the *Gu* and *Si* levels. Logistic regression models were separately fitted for males and females to determine whether exposure to PM_10_ and SO_2_ was associated with the prevalence of DM in different gender groups after adjusting for socio-demographic factors (age, household income, economic activity status, and educational attainment), body mass index (BMI), and smoking behavior. The significance level was set at *P*<0.05.

## Results

### Characteristics of Study Sample and Ambient Air Pollution

The descriptive and summary estimates were assessed, and the differences between male and female groups were compared using Chi-square and t tests (Table 1). Of the 96068 participants 43941 (46%) were males. The mean prevalence of DM among males (6.9%) was 1.1% point higher than that in females (χ^2^=9.921, *P=*0.002). The two gender groups were similar with respect to age. However, the other covariates were significantly different between males and females. The mean household income (t=6.48, *P*<0.001), body mass index (t=70.66, *P*<0.001), the rate of economically active residents (χ^2^ =7812.10, *P*<0.001), and smokers (χ^2^ =49275.87, *P*<0.001) were all significantly higher among males than among females.

**Table 1:** Characteristics of the total population and sub-groups

**Variable**	**Categories**	**Total Population (N=96068)**				**χ^2^ or t**	***P***
		**Male (n=43941)**		**Female (n=52127)**			
		**Mean (Frq)**	**SD (%)**	**Mean (Frq)**	**SD (%)**		
Age (yr)		46.7	15.4	46.7	15.645	0.33	.74
Household income (10,000 KW)		4281.0	2865.2	4159.8	2911.27617	6.48	<0.001
BMI (kg/m^2^)		23.7	2.9	22.4	3.08018	70.66	<0.001
Economic activity	Yes	(34163)	(77.7%)	(26100)	(50.1%)	7812.10	<0.001
	No	(9778)	(22.3%)	(26027)	(49.9%)		
Educational attainment	Elementary>	(3344)	(7.6%)	(8936)	(17.1%)	2300.66	<0.001
	Middle-high	(18185)	(41.4%)	(22158)	(42.5%)		
	College<	(22412)	(51.0%)	(21033)	(40.3%)		
Smoking behavior	Non	(10880)	(24.8%)	(40134)	(94.3%)	49275.87	<0.001
	Former	(13995)	(31.8%)	(1290)	(2.5%)		
	Occasional	(1349)	(3.1%)	(392)	(0.8%)		
	Daily	(17717)	(40.3%)	(1311)	(2.5%)		
Diabetes	Yes	(3043)	(6.9%)	(2868)	(5.5%)	83.64	<0.001
	No	(40898)	(93.1%)	(49259)	(94.5%)		

The descriptive statistics for the measures of exposure to PM_10_ and SO_2_ are presented in Table 2. The annual average of PM_10_ exposure was 44.20 μg/m^3^ (SD: 7.23) and 44.15 μg/m^3^ (SD: 7.11) for males and females, respectively, which were both more than two-fold higher than the annual limit of PM_10_ (20 μg/m^3^) suggested by the WHO (25). The annual average of SO_2_ exposure for male and female groups were 5.40*10^−3^ppm (SD: 1.41) and 5.41*10^−3^ppm (SD: 1.40), respectively. The results of *t*-tests indicated that there was no significant difference (*P*<.05) in PM_10_ and SO_2_ exposure between the gender groups.

**Table 2:** Exposure to PM_10_ and SO_2_ by gender

	**Male**		**Female**		***t*-test**	***P***
	**Mean**	**SD**	**Mean**	**SD**		
PM_10_ (mg/m^3^)	44.20	7.23	44.15	7.11	1.17	.24
SO_2_ (10^−3^ ppm)	5.40	1.41	5.41	1.40	−1.31	.19

### Association between DM and Air Pollution

Tables 3 and 4 show the associations between the prevalence of DM and exposure to PM_10_ and SO_2_, based on the results of the logistic regression models accounting for age, BMI, household income, economic activity status, educational attainment, and smoking behavior.

**Table 3:** Multivariate logistic regression estimates for pm_10_ according to dm, adjusted for age, body mass index, economic activity status, educational attainment, and smoking behavior

**Variable**	**Categories**	**Male (n=43,941)**				**Female (n=52,127)**			
		**OR**	***P***	**95% C.I. for EXP(B)**		**OR**	***P***	**95% C.I. for EXP(B)**	
				**Lower**	**Upper**			**Lower**	**Upper**
Age (yr)		1.070	<0.001	1.066	1.074	1.060	<0.001	1.055	1.064
BMI (kg/m^2^)		1.127	<0.001	1.112	1.143	1.116	<0.001	1.102	1.130
Household income (10000 KW)		1.000	0.690	1.000	1.000	1.000	<0.001	1.000	1.000
Economic activity (vs. yes)	No	1.255	<0.001	1.138	1.384	1.358	<0.001	1.233	1.495
Education attainment (vs. Elementary>)	Middle-High	1.261	<0.001	1.124	1.415	0.699	<0.001	0.634	0.771
	College<	.965	0.603	0.843	1.104	0.336	<0.001	0.280	0.403
Smoking behavior (vs. non-smoker)	Former	1.315	<0.001	1.181	1.465	1.406	0.002	1.132	1.747
	Occasional	1.274	0.078	0.974	1.666	1.457	0.110	.919	2.311
	Daily	1.251	<0.001	1.115	1.405	1.269	0.039	1.012	1.591
PM_10_ (mg/m^3^)		1.003	0.257	0.998	1.008	1.008	0.002	1.003	1.013

**Table 4:** Multivariate logistic regression estimates for SO_2_ according to dm, adjusted for age, body mass index, economic activity status, educational attainment, and smoking behavior

**Variable**	**Categories**	**Male (n=43,941)**				**Female (n=52,127)**			
		**OR**	***P***	**95% C.I. for EXP(B)**		**OR**	***P***	**95% C.I. for EXP(B)**	
				**Lower**	**Upper**			**Lower**	**Upper**
Age (yr)		1.07	<0.001	1.066	1.074	1.059	<0.001	1.055	1.064
BMI (kg/m^2^)		1.127	<0.001	1.112	1.143	1.116	<0.001	1.103	1.130
Household income (10000 KW)		1.000	0.672	1.000	1.000	1.000	<0.001	1.000	1.000
Economic activity (vs. yes)	No	1.255	<0.001	1.138	1.384	1.357	<0.001	1.232	1.494
Education attainment (vs. Elementary>)	Middle-High	1.263	<0.001	1.126	1.417	0.698	<0.001	0.633	0.770
	College<	0.959	0.539	0.838	1.097	0.335	<0.001	0.279	0.401
Smoking behavior (vs. non-smoker)	Former	1.314	<0.001	1.180	1.464	1.400	0.002	1.127	1.740
	Occasional	1.275	0.076	0.975	1.667	1.446	0.117	.911	2.294
	Daily	1.254	<0.001	1.117	1.407	1.269	0.039	1.012	1.591
SO_2_ (10^−3^ ppm)		0.979	0.130	0.952	1.006	1.032	0.026	1.004	1.062

The fitted models indicated that both PM_10_ and SO_2_ were statistically significant factors to predict the prevalence of DM for females: the odds ratio of DM with each 1000 ppm increase in PM_10_ was 1.008 (*P*=0.002), and the odds ratio of DM with each 1000ppm increase in SO_2_ was 1.032 (*P*=0.026) for females.

In contrast, neither of these pollutants was significantly associated with the prevalence of DM among the male population.

Among the control variables, age (*P*<0.01), body mass index (*P*<0.01), and economic activity status (*P*<0.01) were statistically significant factors when predicting the prevalence of DM in both gender groups.

The household income was only significantly related to the prevalence of DM among females. The positive association of the prevalence of DM with daily and former smokers was significant in both gender groups (*P*<0.05).

## Discussion

Seeking to highlight the potential adverse health effect of air pollution on the prevalence of cardiometabolic diseases, we investigated the relationship between DM2 and air pollution in South Korea. The analysis focused on examining gender-related differences in the relationship between DM2 and the two air pollutants, PM_10_ and SO_2_. The reliability of the analysis was enhanced by using nationwide data with a large sample size and air pollution data at the finest grain available in South Korea.

Two major findings were obtained from the analysis. First, exposure to PM_10_ was significantly related to the prevalence of DM2 among women. In contrast, no significant association was apparent in men. Although none of the previous studies on DM2 and PM_10_ (16, 26, 27) reported a gender-dependent relationship, the potential vulnerability of females to PM_10_ has been highlighted elsewhere (14, 28). PM_10_ may be related to the prevalence of DM2 in women by affecting their insulin resistance (14).

Second, exposure to SO_2_ was significantly related to the prevalence of DM2 among women. Unlike PM_10_, little is known about the underlying mechanisms that associate SO_2_ exposure and DM2 in female populations. The inherent gender-related variation may influence the difference in biologic susceptibility between the gender groups (29). Most research that has investigated the relationship between air pollution and DM has focused on the effects of PM and/or NO_2_ on DM2. Exposure to outdoor PM and NO_2_ may have a positive association with DM2 for women (13, 14). Despite some speculation, it remains difficult to provide a clear explanation for the gender-dependent effects of these air pollutants on DM. The results of the present study suggest that SO_2_ may also be considered a risk factor for the DM2, particularly among females.

Another possible explanation for the gender-dependent relationships between DM2 and air pollution is the estimation bias due to exposure measurement error between men and women (13). The present study used air pollution data at finest grain (*Gu*- and *Si*-levels) to minimize the potential measurement error. However, another type of measurement error associated with the intra-urban variation within the scale of a metropolitan area may have been caused by reducing the unit size of exposure measurement. Given that a large portion of daily travels involves intra-urban commuting trips in South Korea (30), it is likely that the exposure of air pollution outside a resident’s neighborhood may have influenced the results.

Men’s daily average travel distance is longer than women’s (31). It is possible that the larger portion of the DM prevalence due to air pollution observed in women will be explained by a lower measurement error for the exposure around the individual’s neighborhood. However, men tend to travel longer distances outside their neighborhood and spend more time there; thus, the effect of the exposure to air pollution around their neighborhoods on the prevalence of DM may be smaller. Further studies are warranted to investigate the possible effect of exposure measurement errors associated with the subjects’ travel patterns.

There are important limitations in the present study. The information on DM and health-related variables in this study was self-reported. The subjectivity that is inherent in self-reporting may have affected the results. Second, it was impossible to consider the affect of the amount of exposure time to air pollution because the cross-sectional design of the study did not allow for collecting information on the subjects’ period of residence in the current neighborhood. Third, the ecological fallacy (32) incurred by using aggregated data of ambient air pollution concentration is a potential concern in the analysis. The measures of air pollution for this study did not necessarily match the exposure experienced by each subject.

Despite these limitations, the results of this study provide further evidence of the relationship between DM and air pollution among Asian populations based on the nationwide data of South Korea. Considering the continuous deterioration of air quality in the northeast Asian region and the Mideast, more research is needed to investigate the potential risks of air pollution on DM. Additionally; further evidence of the gender-dependent connection between air pollution and DM is needed to understand the link between air pollution and DM.

## Conclusion

The commonly encountered levels of PM_10_ and SO_2_ may be associated with DM 2 prevalence in South Korea, but it appears there may be gender differences. In particular, exposure to either PM10 or SO2 was significantly related to the prevalence of DM 2 among women but not among men. While additional research is warranted, specifically to determine investigate the possible effect of exposure measurement errors associated with the subjects’ travel patterns. These findings provide new evidence of an association between air pollution and the risk of diabetes in urbanized areas of South Korea.

## Ethical considerations

Ethical issues (Including plagiarism, informed consent, misconduct, data fabrication and/or falsification, double publication and/or submission, redundancy, etc.) have been completely observed by the authors.
